# Antioxidant Activity and Total Phenols from the Methanolic Extract of *Miconia albicans* (Sw.) Triana Leaves

**DOI:** 10.3390/molecules16119439

**Published:** 2011-11-10

**Authors:** Laís Goyos Pieroni, Fernanda Mendes de Rezende, Valdecir Farias Ximenes, Anne Lígia Dokkedal

**Affiliations:** 1 Biology Department, Faculty of Sciences, Unesp – Univ Estadual Paulista, c.p. 473, 17033-360 Bauru, SP, Brazil; 2 Institute of Biosciences, USP – Univ of São Paulo, c.p. 11416, 05422-970 São Paulo, SP, Brazil; 3 Chemistry Departament, Faculty of Sciences, Unesp – Univ Estadual Paulista, c.p. 473, 17033-360 Bauru, SP, Brazil

**Keywords:** antioxidant activity, flavonoids, *Miconia albicans*, total phenolic contents

## Abstract

*Miconia* is one of the largest genus of the Melastomataceae, with approximately 1,000 species. Studies aiming to describe the diverse biological activities of the *Miconia* species have shown promising results, such as analgesic, antimicrobial and trypanocidal properties. *M. albicans* leaves were dried, powdered and extracted to afford chloroformic and methanolic extracts. Total phenolic contents in the methanolic extract were determined according to modified Folin-Ciocalteu method. The antioxidant activity was measured using AAPH and DPPH radical assays. Chemical analysis was performed with the *n*-butanol fraction of the methanolic extract and the chloroformic extract, using different chromatographic techniques (CC, HPLC). The structural elucidation of compounds was performed using 500 MHz NMR and HPLC methods. The methanolic extract showed a high level of total phenolic contents; the results with antioxidant assays showed that the methanolic extract, the *n*-butanolic fraction and the isolated flavonoids from *M. albicans* had a significant scavenging capacity against AAPH and DPPH. Quercetin, quercetin-3-*O*-glucoside, rutin, 3-(*E*)-*p*-coumaroyl-α-amyrin was isolated from the *n*-butanolic fraction and α-amyrin, *epi*-betulinic acid, ursolic acid, *epi*-ursolic acid from the chloroformic extract. The results presented in this study demonstrate that *M. albicans* is a promising species in the search for biologically active compounds.

## 1. Introduction

Over the past decades, there has been growing interest in plants as new therapeutic agents. Among the substances studied that have biological effects are phenolic compounds. Phenolic compounds comprise the main group of antioxidant substances of vegetable origin and, among these, flavonoids are the most important group [1].

Currently there is much interest in the study of antioxidants because, even in small quantities, they present high therapeutic potential in the prevention and treatment of diseases caused by free radicals [2]. Free radicals found in oxygen or, less specifically, reactive oxygen species (ROS), are products of the normal metabolism of cells. They are associated with the production of energy, phagocytosis, regulation of cellular growth, inter-cellular signaling, and the synthesis of important biological substances. However, in excess, these substances present damaging effects, such as the peroxidation of membrane lipids, damage to the proteins found in tissues and membranes, and damage to enzymes, carbohydrates, and DNA [3]. Because of this, free radicals are associated with a series of pathologies, such as arthritis, hemorrhagic shock, cardiac disease, cataracts, cognitive disfunctions, aging, and cancer, and may be considered the cause or the aggravating factor to the disease conditions [4]. These observations have encouraged the search for new chemical substances of plant origin, especially flavonoids, with antioxidant potential. Therefore, the antioxidant properties of flavonoids have garnered attention for use in preventive nutrition, in the conservation of foods and other substances against oxidative stress, and for contributing to the prevention of chronically degenerative diseases and other pathologies, such as cardiovascular disease and cancer [5].

The Melastomataceae are predominantly pantropical plants, including approximately 163 genera and 4,300 species [6]. *Miconia* is one of the largest genus, including approximately 1,000 species; in Brazil, there are approximately 250 species [7] in forests and savannas, where *Miconia stenostachya* and *M. albicans* prevail [8]. Studies aiming to describe the diverse biological activities of the *Miconia* species have shown promising results. Some studies have described the analgesic effects of crude extracts (hexane, methylene chloride and ethanol) obtained from *Miconia* species [9]. Triterpenes (ursolic acid, oleanolic acid and gypsogenic acid) of *Miconia fallax* DC. and *Miconia stenostachya* (Schrank and Mart.) DC. were active against blood trypomastigote forms of *Trypanosoma cruzi * [10]*.* Phenolic compounds from *Miconia myriantha* Benth. showed inhibitory effects against aspartic proteases secreted by *Candida albicans * [11]. The methanolic extracts of *Miconia albicans*, *Miconia cabucu*, *Miconia rubiginosa*, *Miconia stenostachya* and the chloroformic extract of *M. albicans* were evaluate, *in vivo*, to genotoxic and mutagenic effects using the comet assay and micronucleus test. Their possible protective effects were also evaluated in experiments associating the plant extracts with cyclophosphamide (CPA). All the extracts induced alterations in DNA migration (comet assay); all extracts showed a protective effect against CPA in both assays [12].

The following study presents the quantification of total phenolic contents for the methanolic extract of *M. albicans* leaves as well as an evaluation of the antioxidant properties of this extract, of its *n*-butanolic fraction and of the isolated substances. It also describes the isolation, purification, and structural elucidation of the compounds isolated by the methanolic and chloroformic extracts.

## 2. Results and Discussion

### 2.1. Total Phenols

Total phenolic (TP) contents was expressed in GAE equivalents (mg gallic acid/g dry extract) using the equation based on the gallic acid calibration curve: y = 0.0022x + 0.051 (R^2^ = 0.9978), where y is absorbance at 760 nm and x is gallic acid concentration in mg/L. The results obtained in this study revealed that the number of phenol compounds present in the methanolic extract of *M. albicans *leaves was significant, accounting for 70.04 ± 0.12 mg GA/g dry extract.

### 2.2. Antioxidant Assays

**Figure 1:**
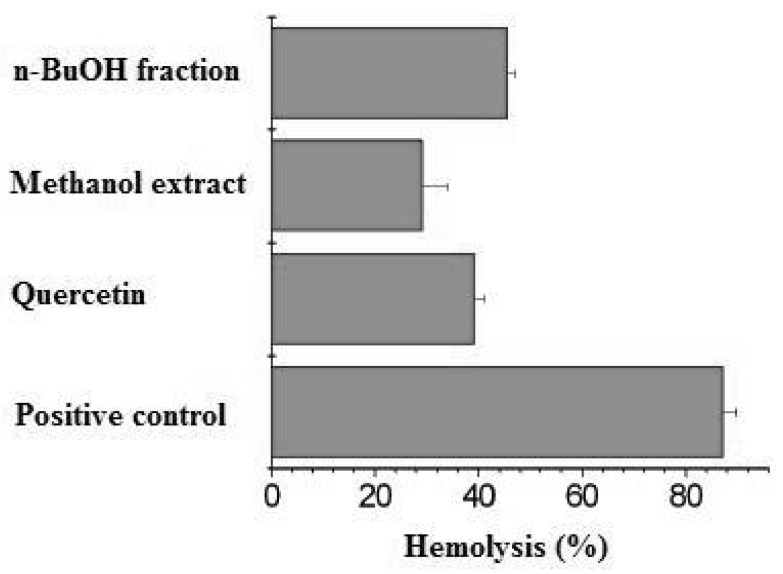
AAPH-induced hemolysis and the protective effect of *n*-BuoH fraction and methanolic extract of leaves from *M. albicans*. Concentration: *n*-BuOH fraction 0.59mg/mL; methanolic extract 4.3mg/mL; quercetin 0.08mg/mL. The reaction mixture (positive control) contained 10% (v/v) erythrocytes and 100 mM AAPH in PBS at 37 °C.

Thermolysis of the azo compound AAPH, which generates a low but constant flux of peroxyl radicals when incubated at 37 °C, provokes hemolysis [13]. The hemolysis of erythrocytes has been extensively used as an *ex vivo* model in the study of ROS-induced disruption of cell membranes. The inhibitory property of many antioxidants upon AAPH-induced hemolysis is well-documented [14,15,16]. Figure 1 shows the protective effect of *n*-butanol fraction and methanolic extract from *M. albicans*, using quercetin as standard substance.

The test using the stable radical DPPH measures a substance’s ability to donate hydrogen radicals to this radical, therefore, the larger the number of hydroxyls present in the sample, the greater its antioxidant activity. The stability of the radical formed is another factor that influences antioxidant potential [17]. The results of the antioxidant activity determined by the DPPH assay showed that the amount of the sample needed to decrease the initial DPPH concentration by 50%, IC_50_ (Table 1), varied from 2.97 ± 0.0105 to 49.45 ± 0.0050 μg/mL. Compound **3** (IC_50_ = 2.97 ± 0.0105 μg/mL) demonstrated the same order of magnitude as the standards, rutin (IC_50_ = 2.55 ± 0.0075 μg/mL) and quercetin (IC_50 _ = 1.60 ± 0.0450 μg/mL), while the MeOH extract presented the highest value (IC_50_ = 49.45 ± 0.0050 μg/mL).

**Table 1:** Antioxidant activity (IC_50_) of the methanolic extract, *n*-butanolic fraction, and isolated flavonoids of *M. albicans*.

Compound	IC_50_ (µg/mL) ± SD
**1 **	14.94 ± 0.0040
**2 **	5.93 ± 0.0035
**3 **	2.97 ± 0.0105
*n*-BuOH Fraction	7.72 ± 0.0350
MeOH Extract	49.45 ± 0.0050
Standard (rutin)	2.55 ± 0.0075
Standard (quercetin)	1.60 ± 0.0450

IC = concentration in µg/mL capable of reducing DPPH by 50%; SD = standard deviation.

### 2.3. Identification of Compounds

A chemical analysis of the methanolic extract was performed to identify the substances responsible for the activities found in the total phenol assays and for the antioxidant activity. Compounds **1**–**4** were isolated from the *n*-butanolic fraction obtained from the methanolic extract. In order to isolate and identify other *M. albicans* compounds, the CHCl_3_ extract was fracionated and compounds **5**–**8** were isolated and identified.

**Figure 2:**
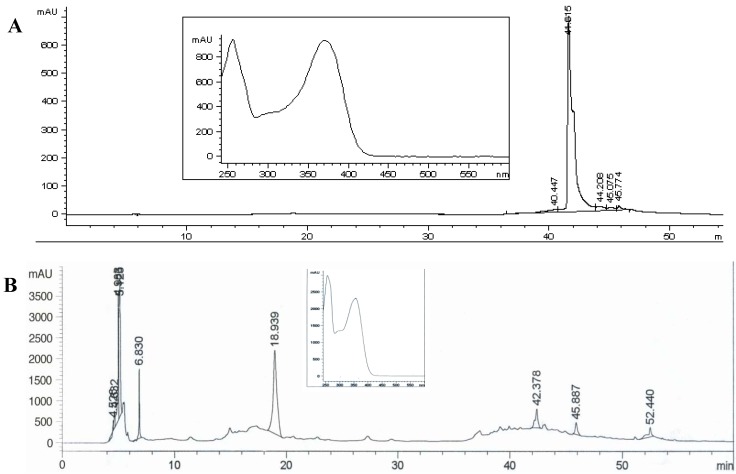
HPLC-DAD chromatograms and respective UV spectrum of compounds **2** (**A**; R_t_ = 18.939 min) and **3** (**B**; R_t_ = 41.615 min). Zorbax SB C_18_ column (250 × 4.6 mm, 5 mm). Solvent gradient: 0–5 min 12% de acetonitrile (B) in acetic acid 0.1% (A); 5–8 min 12–20% B in A; 8–28 min 20% B in A; 28–38 min 20–50% B in A; 38–48 min 50–65% B in A; 48–50 min 65–100% B in A; 50–55 min 100% B; 55–56 min 100–12% B in A; 56–60 min 12% B in A; flows: 0–50 min, 0.5 mL.min^−1^; 50–55 min, 1 mL.min^−1^ and 56–60 min, 0.5 mL.min^−1^; λ = 352 nm.

The ^1^H-NMR of compound **1** showed a typical flavonol spectrum with two doublets overlayed at δ 7.57, related to the H-2’/H-6’ (*J* = 2.0 and 8.0 Hz), a doublet at δ 6.83 (*J* = 8.0 Hz, H-5’), a singlet at δ 6,39 (*J* = 2.0 Hz, H-8), other singlet at δ 6.18 (*J* = 2.0 Hz, H-6) and a doublet at δ 5.45 (*J* = 7.5 Hz, H-1’’). The ^13^C-NMR spectrum showed 15 carbons related to the flavonol moiety and six additional signals indicating the presence of a sugar moiety. Thus, the compound was identified as quercetin-3-*O*-glycoside. Compound **2** (R_t_ = 18.939 min) was identified as rutin by HPLC-DAD after comparison with data in the equipment’s database. Compound **3** (R_t_ = 41.615 min) were identified as quercetin in comparison with the same database. Figure 2 shows the HPLC chromatograms of compounds **2** and **3**.

The ^1^H-NMR spectrum of compound **4** suggested the presence of a *p-*coumaroyl moiety since the ^1^H showed peaks at δ 6.37 (1H, *d*, *J *= 16.0Hz), δ 7.51 (1H, *d*, *J* = 16.0Hz), δ 6.78 (2H, *d*, *J* = 8.0Hz), δ 7.49 (2H, *d*, *J* = 8.0Hz). The ^1^H-NMR spectrum also contained signals due to eight tertiary methyl groups (δ_H_ 0.70, 0.79, 0.81, 0.85, 0.88, 0.89, 0.92 and 1.03) and one olefinic proton (δ_H_ 5.13), which are typical of α-amyrin. Additionally the ^13^C-NMR data for this compound were very similar to those of a ursane-type triterpene related to α-amyrin [18], except for C-2, 3 and 4 (Table 2). These results indicate that **4** is α-amyrin bearing a *p-*coumaroyl ester moiety at the C-3 position. Thus, this compound was determined to be 3-(*E*)-*p*-coumaroyl-α-amyrin.

The ^1^H-NMR spectrum of compound **5** exhibited five methyl singlets and two methyl doublets of a methyl ursolate. The ^13^C-NMR spectrum of this compound showed the peak corresponding to C-3 at δ 79.1, typical of β-hydroxyl group and peaks relative to C-12 and C-13 at δ 124.4 and δ 139.6, respectively, typical of ursane derivatives. According to the literature, this compound was identified as α-amyrin.

The analysis of the ^1^H-NMR spectrum of **6** revealed a typical lupane skeleton. This spectrum was characterized by signals for five tertiary methyls (δ 0.91–0.63, Me-23–Me-27) and one vinylic methyl (δ 1.68, Me-30) and two protons of a isoprenyl moiety at δ 4.54 and 4.65 (1H each, d, *J* = 2.0 Hz, H_a_-29, H_b_-29). The Δ^20,29^-functionality of a lupene skeleton was inferred for this compound from the ^13^C spectra, that revealed the resonances of the sp^2^ carbon at C-29 at δ 109.6 and C-20 (quaternary carbon) at δ 150.4. The peak relative to C-3 at δ 76.9 suggests a OH group in α- position. Therefore, **6** were deduced as *epi*-betulinic acid.

**Figure 3:**
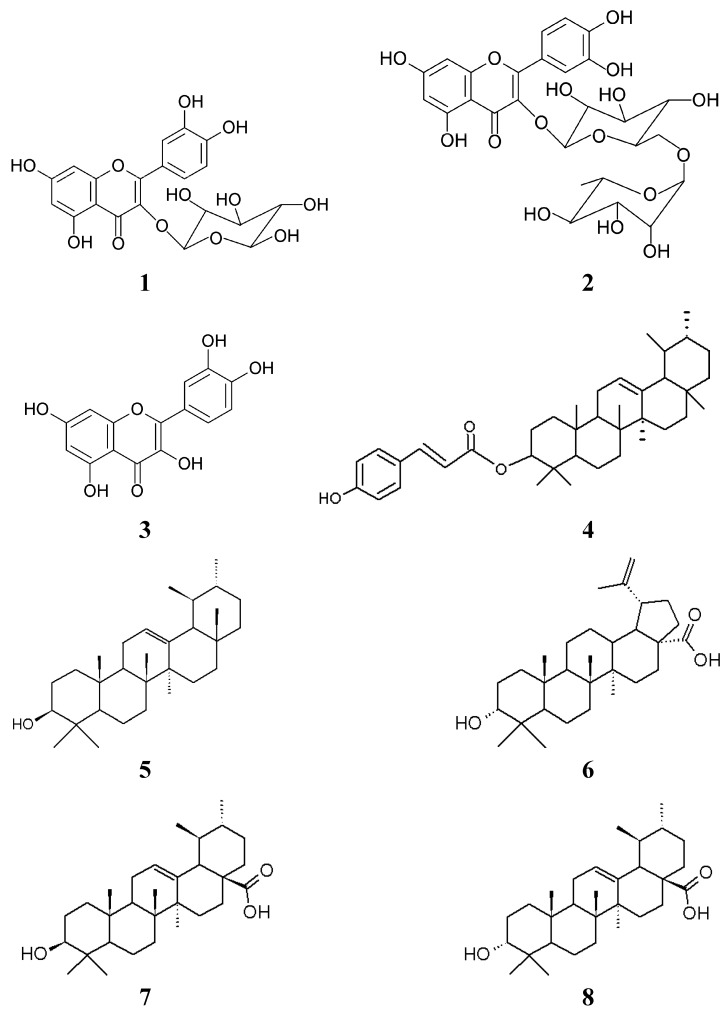
Isolated substances from *M. albicans.*

The ^1^H-NMR spectra of compounds **7** and **8** were very similar, exhibiting five methyl singlets and two methyl doublets of a methyl ursolate. The ^13^C-NMR spectra of the two compounds confirmed the ursane skeleton, with the peak relative to C-3 at δ 79.3 (**7**) and δ 76.9 (**8**), suggesting a OH group in β- and α- position, respectively. Peaks relative to C-12 and C-13 are seen at δ 126.1 and δ 139.8 (compound **7**) and at δ124.6 and δ138.2 (compound **8**). Compound **7** was identified as ursolic acid and compound **8 **was identified as *epi*-ursolic acid. The attributions of all compounds could be seen in Table 2 and the structure of each compound are present in Figure 3.

**Table 2:** ^13^C-NMR data for compounds **4**–**8** from *M. albicans* (125MHz, DMSO-d_6_) ^a^.

Carbon	4	5	6	7	8	
1	38.2	38.8	34.0	38.7	36.3	
2	22.8	27.3	25.1	27.5	27.5	
3	82.3	79.1	76.9	79.3	76.9	
4	39.3	38.8	38.5	38.7	38.2	
5	55.0	55.2	54.9	55.2	54.8	
6	17.5	18.3	18.0	18.6	18.1	
7	32.3	32.9	33.9	33.2	32.7	
8	40.4	39.6	41.2	39.1	38.5	
9	46.9	47.7	49.9	47.8	47.0	
10	36.9	36.9	36.7	36.9	36.5	
11	23.2	23.3	20.5	23.5	23.8	
12	125.0	124.4	27.1	126.1	124.6	
13	138.3	139.6	37.6	139.8	138.2	
14	42.0	42.2	42.6	42.2	46.9	
15	28.6	29.7	31.7	28.5	26.9	
16	26.6	26.6	36.4	24.4	26.9	
17	33.7	33.7	55.5	33.2	32.7	
18	55.9	59.1	46.7	52.9	52.4	
19	39.2	39.7	48.7	39.3	39.4	
20	39.2	39.6	150.4	39.3	39.3	
21	32.2	31.2	30.1	30.0	30.2	
22	41.5	41.5	38.3	23.6	23.8	
23	28.4	28.1	28.1	28.2	28.3	
24	16.9	15.6	15.8	15.7	15.2	
25	16.9	15.7	15.9	15.8	16.1	
26	16.8	16.8	16.0	17.0	16.9	
27	23.3	23.4	14.4	23.8	23.3	
28	28.2	28.1	177.3	178.3	178.3	
29	17.4	17.4	109.6	17.3	17.0	
30	21.0	21.3	19.0	21.3	21.1	
28OMe			51.3			
1’	160.0					
3’	145.0					
4’	127.3					
5’	130.0					
6’	116.0					
7’	157.7					
8’	116.0					
9’	130.0					

^a^ Chemical shifts are in ppm.

Considering antioxidant activity and the total phenolic contents, there are several studies that support the thesis that high biological activity is related to the presence of phenolic compounds [19]. The high level of total phenols observed in the *M. albican*s methanolic extract correlates with the best antioxidant activity test results. Among the substances best known for their antioxidant effects are polyphenols and gallates [20]. Flavonoids belong to a group of polyphenols and, generally, are found as glycosides. The flavonoid ability to act as an antioxidant agent in biological systems is perhaps its most important activity and the most studied in the past years. Several epidemiological studies have shown the effects of diets rich in fresh fruits and vegetables against the risk of contracting cardiovascular diseases and certain cancers. These beneficial effects have been attributed, in part, to the presence of phenolic compounds, among these are flavonoids, which effects are the result of their antioxidant properties. The electron donating properties of flavonoids are very well defined and explain the antioxidant activity *in vitro* of these compounds [21]. The results obtained through these assays with antioxidant activity demonstrate that the methanolic extract, *n*-butanolic fraction, and isolated flavonoids of the *M. albicans *present significant free radical scavenging activity.

Many studies on the antioxidant potential of phenolic compounds in plants concluded that it is impossible to predict the antioxidant power of a given product by studying just one type of flavonoid or other kind of antioxidants contained in the sample. In some cases the possible existence of synergic or antagonistic effects between the various antioxidants present in plant has been postulated [22]. The *n*-butanolic fraction presented significant antioxidant activity results in both tests performed, suggesting a synergic effect, related to its flavonoid content.

## 3. Experimental

### 3.1. General

The Folin-Ciocalteu reagent and the sodium carbonate (Na_2_CO_3_) were purchased from Dinâmica, the DPPH (2,2-diphenyl-1-picrylhydrazyl) radical from Aldrich Co., the gallic acid from Vetec, rutin and quercetin from Sigma-Aldrich. Absorbance measurements were taken using a Cintra 10_e_ UV-Visible spectrophotometer to determine the total phenolic contents. A UV-1240 (Shimadzu, Japan) spectrophotometer was used for the AAPH assay and a PowerWave XS BioTek® spectrophotometer was used for the tests with the DPPH radical. The HPLC analyses were performed using a HP series II 1090 liquid chromatogram. NMR spectra in DMSO-d_6_ were obtained using a Varian INOVA 500 spectrometer, operating at 500 MHz for ^1^H and 125 MHz for ^13^C.

### 3.2. Plant Material

The aerial parts (1.2 kg) of *Miconia albicans* (Sw.) Triana were collected in the UNESP campus of Bauru (São Paulo, Brazil) and identified by Dr. Anne L. Dokkedal. A voucher specimen was deposited in the Herbarium of the Biology Department of UNESP/Bauru, under number ALD 145.

### 3.3. Extraction and Isolation

The leaves were dried (at 60 °C for 4 days) and powdered. The dry powdered material was macerated three times with 2 liters of chloroform and methanol successively at room temperature and left for 48 hours in the respective solvent. The solvents were filtered and evaporated at 35 °C under reduced pressure providing CHCl_3_ and MeOH extracts. The extracts obtained were determined as a percentage: 15.0 (180.0 g) and 3.1 (37.20 g) for MeOH and CHCl_3_ extracts of *M. albicans*, respectively. A sample of of MeOH extract (16.0 g) underwent liquid-liquid extraction between *n*-BuOH and H_2_O, affording a residue (10.0 g). An aliquot (3.0 g) was then submitted to gel filtration chromatography on a Sephadex LH-20 (Pharmacia) column (100 cm × 5 cm i.d.) using MeOH as eluent. Fractions (8.0 mL) were collected and analyzed by cTLC [*n*-BuOH/acetic acid/ H_2_O (6:1:2)] and sprayed with anisaldehyde/H_2_SO_4_ solution for visualization. Fraction 78 (6.8 mg) afforded compound **1** that was considered pure and analyzed by NMR. Fractions 64–71 (13.1 mg), 130–142 (11.0 mg) were analyzed by reversed-phase C_18_ HPLC eluted with acetonitrile (A)/acetic acid 0.1% (B) (gradient), afforded compounds **2** and **3**, respectively. The fractions 35–43 (90.0 mg) were submitted to further fractioning in a silica gel column eluted with hexane, chloroform and methanol (gradient). From this fractionation, fraction 11 (15.0 mg) (**4**) was considered pure and analyzed by NMR. The CHCl_3_ extract was submitted to a silica gel column chromatography eluted with hexane, AcOEt and MeOH (gradient). Fractions 25 (78.3 mg) and 77 (83.5 mg) were submitted to pTLC and analyzed by NMR, afforded compounds **5** (13.0 mg) and **6** (9.7 mg), respectively. Two other compounds (**7**, 4.0 mg and **8**, 7.8 mg) were isolated after recrystalization phases with MeOH and analyzed by NMR.

### 3.4. Determination of Total Phenolics

Total phenolic contents in the extract were determined according to modified Folin–Ciocalteu method [23]. An aliquot of the extract (0.5 mL) was mixed with Folin- Ciocalteu reagent (2.5 mL, previously diluted with water 1:10 v:v) and an aqueous Na_2_CO_3_ solution (7.5 mL). The tubes were allowed to stand for 120 min at room temperature for color development. Absorbance was then measured at 760 nm. Samples of extract were evaluated at a final concentration of 0.1 mg/mL. Total phenolic contents were determined by interpolation of absorbance of the samples against a calibration curve made with gallic acid standard (50 to 500 μg/mL) and expressed as mg gallic acid equivalents (GAE) per g of dry extract. The samples were made all in duplicate.

### 3.5. Determination of Antioxidant Activity

#### 3.5.1. Erythrocyte Suspension

Human erythrocytes from healthy donors were obtained from peripheral blood, centrifuged at 1,500 rpm for 10 min, and washed three times with phosphate-buffered saline (PBS) at pH 7.4. The supernatant and buffy coat was removed by aspiration after each wash. The cells were resuspended to 20% (v/v) in PBS. The blood samples were taken from healthy volunteers.

#### 3.5.2. Hemolysis Assays

The hemolysis studies were performed as previously described by Ko *et al.* [24]. Equal amounts of erythrocytes suspension and 200 mM AAPH in PBS were gently homogenized while being incubated for 150 min at 37 °C (blood tube rotator). Aliquots (75 μL) were removed at regular intervals, diluted 1:20 with PBS, and centrifuged at 4,000 rpm for 10 min. The degree of hemolysis was measured in the supernatant by its absorbance at 540 nm. Reference values (100% hemolysis) were determined with the same aliquot of erythrocytes but diluted in 1,500 μL of distilled water instead of PBS to provoke the total lysis of the erythrocytes. By diluting the 100% hemolysis sample in PBS, a calibration curve of hemolysis percentage against absorbance was constructed for conversion of the absorbance measurement to degrees of hemolysis. The blank was PBS. Ten μL aliquots of the methanol extract and the *n-*butanol fraction from *M. albicans *in ethanol were added at the beginning of the reaction. The same volume of ethyl alcohol (10 μL) was added to the negative (without AAPH) and positive (with AAPH) controls.

#### 3.5.3. Determination of Scavenging Activity Against DPPH Radical

This method was based on previously described studies [25]. First, DPPH methanolic solution (50 mL) was prepared at a concentration of 40 μg/mL, and kept refrigerated and protected from light. On hundred μL of solutions containing different concentrations of the methanolic extract, the *n*-butanolic fraction and the isolated compounds diluted in methanol (1.67, 2.67, 3.33, 6.67, 10.0, 33.3 and 66.7 μg/mL) were added to DPPH methanolic solution (200 μL). Rutin and quercetin were used as standards in final concentrations of 1.04, 2.01, 4.09, 8.11, 12.20, 16.29, 20.31 μg/mL and 0.51, 0.99, 2.02, 4.02, 6.04, 8.06, 10.05 μg/mL, respectively. After a 30 min incubation period, the absorbance was measured against a blank (MeOH) at 517 nm. One hundred μL of MeOH added to the DPPH methanolic solution (200 μL) was used as a positive control. The IC_50_ (extract concentration providing 50% inhibition of DPPH) was calculated using the statistical program Origin^®^ 8.1. As such, the lower the IC_50_ value, the higher the antioxidant activity within the sample. All samples were carried out in triplicate.

## 4. Conclusions

Considering that natural substances may be responsible for the protective effect against the risks for several pathological processes, the results presented in this study demonstrate that *M. albicans* is a promising species in the search for biologically active compounds.
